# Reducing Uncertainty in Within-Host Parameter Estimates of Influenza Infection by Measuring Both Infectious and Total Viral Load

**DOI:** 10.1371/journal.pone.0064098

**Published:** 2013-05-15

**Authors:** Stephen M. Petrie, Teagan Guarnaccia, Karen L. Laurie, Aeron C. Hurt, Jodie McVernon, James M. McCaw

**Affiliations:** 1 Melbourne School of Population and Global Health, The University of Melbourne, Parkville, Victoria, Australia; 2 Monash University, Churchill, Victoria, Australia; 3 World Health Organization Collaborating Centre for Reference and Research on Influenza, North Melbourne, Victoria, Australia; 4 Vaccine and Immunisation Research Group, Murdoch Childrens Research Institute, Royal Childrens Hospital, Parkville, Victoria, Australia; University of Georgia, United States of America

## Abstract

For *in vivo* studies of influenza dynamics where within-host measurements are fit with a mathematical model, infectivity assays (e.g. 50% tissue culture infectious dose; TCID_50_) are often used to estimate the infectious virion concentration over time. Less frequently, measurements of the total (infectious and non-infectious) viral particle concentration (obtained using real-time reverse transcription-polymerase chain reaction; rRT-PCR) have been used as an alternative to infectivity assays. We investigated the degree to which measuring both infectious (via TCID_50_) and total (via rRT-PCR) viral load allows within-host model parameters to be estimated with greater consistency and reduced uncertainty, compared with fitting to TCID_50_ data alone. We applied our models to viral load data from an experimental ferret infection study. Best-fit parameter estimates for the “dual-measurement” model are similar to those from the TCID_50_-only model, with greater consistency in best-fit estimates across different experiments, as well as reduced uncertainty in some parameter estimates. Our results also highlight how variation in TCID_50_ assay sensitivity and calibration may hinder model interpretation, as some parameter estimates systematically vary with known uncontrolled variations in the assay. Our techniques may aid in drawing stronger quantitative inferences from *in vivo* studies of influenza virus dynamics.

## Introduction

Influenza is an infectious disease that causes significant morbidity and mortality worldwide [2]. Human influenza infection is usually localised to the upper respiratory tract (URT) [1], and generally lasts for approximately one week [1], [3]–[5]. Mathematical modelling of *in vivo* or *in vitro* influenza experiments can be used to improve our understanding of the dynamics of infection [6]–[8], and to subsequently provide useful insights into areas such as: the assessment and optimisation of antiviral drug treatment strategies [4], [9], the assessment of relative fitness between different influenza strains [10], and the optimisation of vaccine production [11], [12]. Recent reviews of mathematical modelling of influenza infection have highlighted the need for more precise, comprehensive datasets in order to generate more reliable estimates of the parameters that govern infection dynamics [7], [8].

For *in vivo* studies of within-host influenza dynamics, infectivity assays such as 50% tissue culture infectious dose (TCID_50_) or plaque assays are often used as a measure of the *infectious* (viable) virion concentration over time [3]–[5], [13]–[19] – we define infectious virions to be virions that can infect susceptible cells and initiate the production of progeny virus. In addition to infectious virions, infected cells can also produce non-infectious viral particles [20], [21]. In some *in vivo* influenza modelling studies [15], [22]–[24], real-time reverse transcription-polymerase chain reaction (rRT-PCR) assays that quantify viral RNA (vRNA) have been used as an alternative to infectivity assays – we define *total* (infectious and non-infectious) viral particles to be particles that contain vRNA measurable via rRT-PCR. Mathematical models that have been fitted to such total viral load data have implicitly assumed that the proportionality between infectious and total viral particle concentration is constant over time.

However, in an *in vitro* influenza study, Schulze-Horsel *et al.* [12] employed TCID_50_ and hemagglutination (HA) assays as quantifications of infectious and total viral load, respectively. They fitted these data using a mathematical model that included both infectious and non-infectious viral particles, and found that infectious particles decayed faster than non-infectious particles. Similarly, results from previous influenza studies have suggested that the *in vivo* ratio of infectious to total viral particles changes over time (e.g. [25]–[28]; reviewed in [7]), and this has also been suggested by results obtained for other viruses [29]–[32]. Recently, in an *in vitro* study, Iwami *et al.* [33] used both TCID_50_ and rRT-PCR assays and a corresponding mathematical model to draw improved inferences on the replication kinetics of simian/human immunodeficiency virus (SHIV).

Here we investigate *in vivo* whether measurement of both infectious and total influenza virus, when fit with an appropriate within-host model, can reduce uncertainties when estimating model parameters. We develop a mathematical model of influenza infection in ferrets, based on previous *in vitro* [12], [33] and *in vivo* [3] models, and fit it to TCID_50_ and rRT-PCR data from experiments performed by Guarnaccia *et al.* (under review). We find that measurement of both infectious (via TCID_50_) and total (via rRT-PCR) viral particle concentration allows some within-host model parameters to be estimated with reduced uncertainty – and with greater consistency in best-fit values across different experiments – when compared with parameter estimates obtained from fitting to infectious viral load data alone.

## Methods

### Ethics Statement

All ferret experiments were conducted with approval from the CSL Limited/Pfizer Animal Ethics Committee, in accordance with the Australian Government, National Health and Medical Research Council, Australian code of practice for the care and use of animals for scientific purposes (license number: SPPL 051).

### Ferret Experimental Data

We analyse viral load data taken from an experiment performed by Guarnaccia *et al.* (under review). This study investigated the likelihood of an antigenically drifted mutant virus arising during serial passages of a wild-type A(H1N1) 2009 pandemic virus (A/Tasmania/2004/2009) through ferrets. We analyse data obtained from the two control groups used in this study – one in which ferrets were immunised with only an adjuvant prior to infection with the challenge virus (designated “PBS+IFA”), and another in which ferrets received no immunisation (designated “Naïve”). Two separate serial passage lines (A and B) were run for each group.

Each serial passage line included eight “naturally-infected” ferrets (designated R0–R7). Each of these ferrets were infected by being co-housed with the preceding ferret in the serial passage line (or in the case of each R0 ferret, with an infected ferret that had received no immunisation). Each infected ferret was co-housed with the next ferret in line only once the infected ferret had attained a high enough viral load (assessed via either rRT-PCR or a rapid influenza test) that the authors believed it was likely to be infectious.

All ferrets were nasal washed daily throughout the experiment. Total vRNA concentration within these samples was measured using rRT-PCR assays (by amplification of influenza A matrix 1 gene). Infectious viral load was measured for multiple samples at a time by performing TCID_50_ assays in batches (see “Fitting the data”). Although rRT-PCR assays were standardised using RNA standards, TCID_50_ assays did not include internal standards for inter-assay calibration.

We fit within-host models separately to each of the following four datasets:

1. Naive line A, ferrets R0–R7,
2. Naive line B, ferrets R0–R7,
3. PBS+IFA line A, ferrets R0–R7,
4. PBS+IFA line B, ferrets R0–R7.

For datasets 1 and 2, rRT-PCR was used to determine the time that each ferret was co-housed with the next ferret in the serial passage line, while the rapid test was used for datasets 3 and 4.

### Within-host Models

#### Single-measurement model

**Figure 1:**
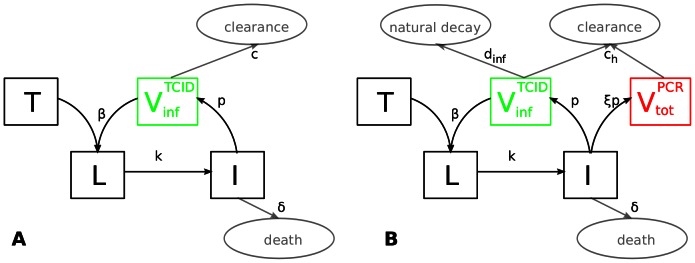
Within-host models. Schematics of (**A**)**:** the single-measurement model, where 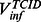 is fit to TCID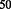 data, and (**B**)**:** the dual-measurement model, where 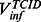 and 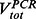 are fit to TCID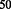 and rRT-PCR data, respectively. For clarity, the colours of the 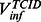 and 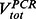 compartments (green/red) are matched to the colours of those compartments in Figure 2.

Here we outline a model of the ferret upper respiratory tract (URT) that we fit solely to TCID_50_ measurements; the “single-measurement” model (Figure 1A). In this model, free infectious virions (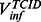) infect susceptible epithelial cells (“target” cells, 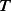) at the rate 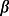, producing latently infected cells (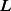). These latent cells become productively infected cells (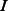) at the rate 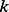, which in turn produce infectious virions at the rate 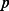 and undergo cell death at the rate 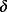. Infectious virions are cleared at the rate 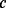. The units of all state variables and parameters in this model are shown in Table 1. The system of ordinary differential equations (ODEs) that govern the dynamics of this model as a function of time 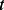, are:
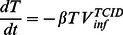
(1)

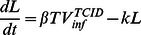
(2)

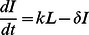
(3)

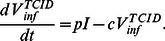
(4)


**Table 1:** Single-measurement model variables.

	Description	Units
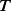	number of target cells	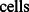
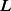	number of latently infected cells	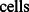
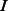	number of productively infected cells	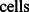
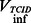	concentration of free infectious virions measured via TCID_50_ infectivity assay	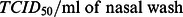
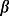	rate governing infection of target cells by infectious virions	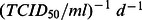
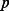	rate of production of infectious virions	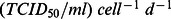
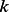	transition rate from latent to productive infection	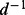
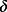	death rate of productively infected cells	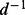
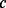	clearance rate of infectious virions	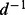

Definitions of all state variables (compartments) and parameters in the single-measurement model.

This model and similar extensions, have been used previously to simulate both *in vivo* and *in vitro* influenza dynamics [3], [5], [9], [15], [19], [23], [34].

The system of ODEs given in Equations 1–4 implicitly assumes an exponential distribution for the times spent by cells in each of the latent (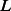) and infected (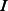) states. However, models with normal or lognormal distributions for 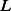 and 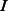 lifespans have been found to produce more accurate fits to *in vitro* data (single-cycle viral yield experiments), compared with models that employ exponential distributions or fixed delays [5], [34]. We model more biologically realistic distributions for the 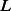 and 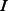 lifespans (without increasing the number of parameters) by employing the method of stages [35], whereby each of the 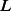 and 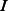 compartments is split into several subcompartments or stages. We include 20 stages within each compartment, as this produces standard deviations for the distributions of the 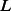 and 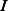 lifespans that are consistent with *in vitro* estimates and fixed values from previous studies [10], [34], [36].

We fit the 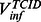 state in the resulting single-measurement model to TCID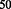 viral load data, noting as others have [4], [7]–[9], that the units of an infectivity assay may underestimate the actual concentration of infectious virions at the site of infection.

#### Dual-measurement model

Here we extend the single-measurement model to include an additional state variable for total viral particles (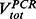). This compartment incorporates both infectious virions and non-infectious viral particles (which contain vRNA but are not capable of infecting susceptible cells). Infected cells produce both types of viral particles in this “dual-measurement” model (Figure 1B). We fit model output from 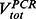 to rRT-PCR measurements, and fit 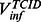 to TCID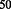 measurements.

In order to derive this model, we must build upon a “biological” model that explicitly incorporates counts of actual numbers of infectious virions (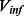) and non-infectious viral particles (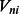). Similar to other modelling studies (e.g. [33]), we introduce scaling relationships:
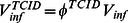
(5)

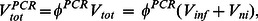
(6)that transform the biological model, so that “rescaled” viral load compartments (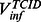 and 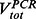) can be fitted directly to assay data. We then make the following assumptions: (1) the ratio of non-infectious to infectious particles produced by infected cells is constant over time (assumed in previous *in vitro* modelling studies [12], [33], [36]); (2) the host’s immune response clears both infectious and non-infectious particles at the same rate, 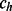 (assumed in several models of human immunodeficiency virus infection [37], [38]); and (3) this host-driven clearance rate is much larger than the degradation rate of non-infectious particles, 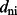 (the biological plausibility of this assumption is supported by comparing previous estimates of the viral clearance rate and viral degradation rate; see Text S1). Under these assumptions we obtain the dual-measurement model:



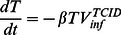
(7)

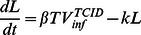
(8)

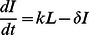
(9)

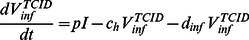
(10)

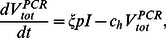
(11)where total viral particles are produced from infected cells at the rate 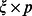 (thus 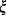 gives the ratio of total vRNA measured via rRT-PCR to infectious virions measured via TCID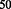, as produced by infected cells), and infectious virions degrade into non-infectious viral particles at the rate 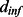. This degradation process does not change the concentration of vRNA, and hence does not change the concentration of 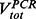. In the single-measurement model, degradation of infectivity was implicitly incorporated into the clearance rate, 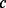. The 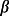, 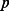, and 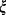 parameters are related to their corresponding biological model parameters (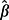, 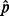, and 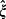) via: 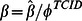, 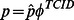, and 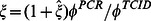.

The units of all state variables and parameters in the dual-measurement model are shown in Table 2. In order to produce more biologically accurate distributions for the latent and infected cell lifespans, we again split the 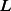 and 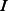 compartments into 20 stages as outlined above in the context of the single-measurement model.

**Table 2:** Dual-measurement model variables.

	Description	Units
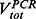	concentration of total vRNA (from infectious and non-infectious free viral particles) measured via rRT-PCR assay	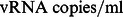
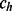	host-driven clearance rate (assumed to be the same for both infectious and non-infectious viral particles)	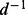
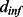	rate of degradation of infectious virions to non-infectious viral particles	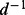
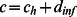	clearance rate of infectious virions	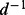
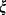	ratio of total vRNA measured via rRT-PCR to infectious virions measured via TCID_50_, as produced by infected cells	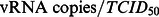

Definitions of all state variables (compartments) and parameters in the dual-measurement model that do not appear in the single-measurement model (Table 1).

### Fitting the Data

We estimate the best fit for a given model by performing a nonlinear least squares fit to viral load data in log-space. For the dual-measurement model, the sum of squared residuals (SSR) for a single set of model parameters (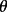) is given by:
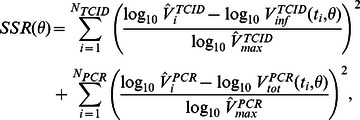
(12)where 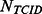 and 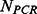 are the number of TCID_50_ and rRT-PCR data points being fitted, respectively, 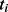 is the time that the 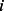
th data point was measured, 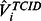 and 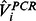 are the 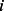
th TCID_50_ and PCR measurements, respectively, and 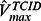 and 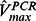 are the maximum values that were obtained across all TCID_50_ and PCR measurements, respectively. The weighting of each respective term by 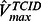 and 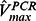 makes the corresponding residuals dimensionless. This is similar to the weighting procedure that Saenz *et al.* [22] used to fit a within-host influenza model to *in vivo* measurements of viral load, innate immune response, and the cumulative proportion of cells that become infected and die over the course of infection.

For any measurement of (infectious or total) viral load, the actual concentration may be below the relevant assay's detectability threshold, in which case assay results provide only an upper bound on the state. We will refer to this as a “non-detection”. Further, the TCID_50_ assay may also saturate (a “max-detection”), due to a limited number of available wells in the assay. Such max-detections provide only a lower bound on the infectious viral load. For any non-detection data points, if the relevant (infectious or total) viral load state in the model is above the detectability threshold at that timepoint, then we calculate the relevant (TCID_50_ or PCR) term of the SSR as per Equation 12. However, if the simulated viral load concentration lies below the detectability threshold at that timepoint then, as in [22], we do not include any contribution to the relevant term of the SSR. Similarly, for any max-detection data points, we include a non-zero contribution to the TCID_50_ term of the SSR only if 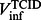 is below the maximum threshold at that timepoint – otherwise, no contribution to the SSR is made.

When fitting the single-measurement model to data, we use the SSR in equation 12 with the PCR term omitted. For both models, the SSR is minimised using MATLAB R2011b’s genetic algorithm to perform global optimisation.

For any naturally-infected ferret, the time when infection actually occurred is unknown. We assume that infection occurred 24 hours before the first positive (above-threshold) viral load measurement was taken for each ferret. We define this time to be 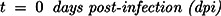 and run all model simulations from this point onwards. For 31 of the 32 ferrets in datasets 1–4, 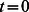 matches up with the time that each ferret was co-housed with the previous ferret in the serial passage line (the only ferret where this is not the case is the N2 ferret in dataset 1, where 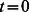 corresponds to 24 hours after co-housing began).

We use the following initial conditions when fitting each model to data:

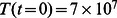
 (an estimate of the number of epithelial cells in the ferret URT; based on an estimate of 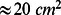 for the URT surface area of mammals that are similar in size to ferrets [39], and an estimated surface area per ferret epithelial cell of 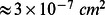, which is similar to previous estimates of epithelial cell surface area for both humans [3] and mice [16]),
- *L*(0) = *I*(0) = 0,

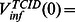
 fitted parameter (this parameter can be interpreted biologically as the initial infectious viral inoculum, but only when ferrets were indeed infected at 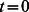).

For the dual-measurement model, we define 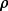 to be the ratio of total to infectious free viral load:
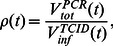
(13)so that the initial total viral concentration is given by 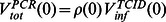, where 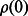 is a fitted parameter. When fitting each model to data, we fix 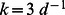 and estimate all other parameters (see Text S1 for more detail, including the biologically realistic ranges that we use to constrain parameter estimates).

We determine uncertainties in parameter estimates by plotting likelihood confidence regions (LCRs) in parameter space [40] and estimating parameter confidence intervals (CIs) [41]. LCRs provide good approximations of confidence regions for nonlinear models [40], [41], and the LCR method has been shown to estimate confidence regions and confidence intervals more reliably than linearisation methods [41]. LCRs can be generated during the genetic algorithm optimisation procedure, at the 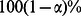 confidence level, by plotting all parameter sets that have a corresponding SSR value that satisfies [40]:
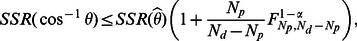
(14)where 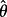 is the best-fitting (optimal) parameter set, 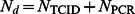 is the total number of data points being fitted, 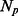 is the number of unknown model parameters, and 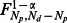 is the 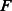-distribution with 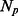 and 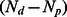 degrees of freedom at the 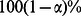 confidence level. Confidence intervals (CIs) for each parameter are given by the projection of the LCR onto that parameter’s axis [41].

Lastly, TCID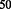 assays have an inherent variability that can systematically shift all assay results towards higher or lower 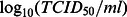 concentrations. Three different TCID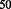 assays were performed: (i) dataset 1, (ii) dataset 2, and (iii) datasets 3 and 4. It can be shown that a shift in log-space by some factor – say 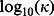 – would rescale certain parameter estimates. For both models, 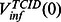, 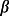, and 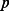 are rescaled according to:
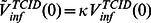
(15)

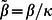
(16)

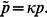
(17)


For the dual-measurement model, 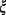 is also rescaled:
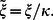
(18)


Beauchemin *et al.* [7], [9] performed a similar analysis to this when investigating the effects of measurement variability on model parameters.

## Results

### Fits to Viral Load Data

**Figure 2:**
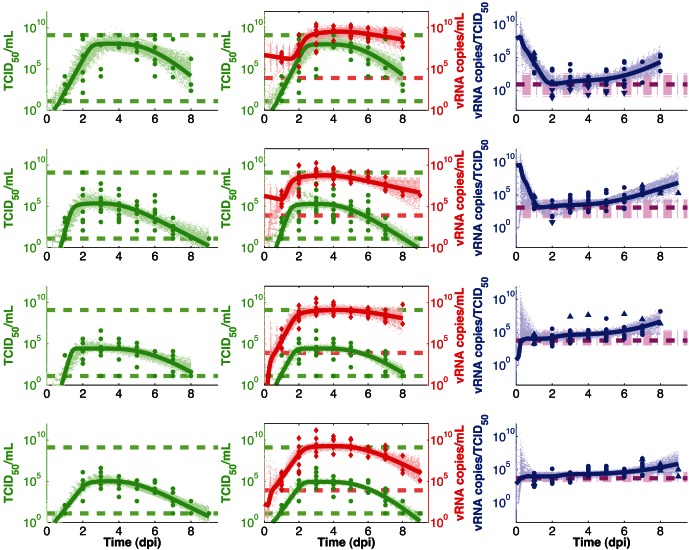
Best fits to viral load data. Combined viral load data from all ferrets are shown for datasets 1 (top row) to 4 (bottom row). For the single-measurement model (left column), we show the best-fit of infectious viral load (solid green line; 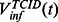) to TCID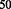 data (green dots; dashed green lines give lower and upper thresholds; some dots overlap as there are occasionally multiple data points at exactly the same TCID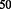 level). For the dual-measurement model (centre and right columns), we show the best-fits of infectious (solid green line) and total (solid red line; 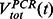) viral load to TCID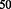 data (green dots) and rRT-PCR data (red diamonds; dashed red line gives lower threshold), respectively. We also show the ratio of rRT-PCR to TCID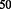 data (blue dots), as well as the 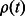 curve (solid blue line) and 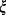 value (solid mauve line) generated by the best-fit of the dual-measurement model. Whenever a TCID_50_ measurement is a non-detection (lower threshold) or max-detection (upper threshold), the corresponding 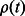 measurement is a lower limit (upward-pointing blue arrows) or an upper limit (downward-pointing blue arrows), respectively. In addition to the best-fit lines, we also plot 500 randomly sampled fits with SSR values that satisfy Equation 14 at the 95% confidence level (faded dotted lines for 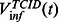, 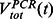, and 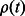, and faded dot-dashed lines for 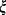).

Fits of each model to combined viral load data, for each of the four different ferret experiments, are shown in Figure 2. We fit the single-measurement model solely to TCID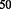 data, while the dual-measurement model is fit to both TCID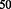 and rRT-PCR data. The ratio of rRT-PCR to TCID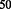 data is not constant over time. This reflects the fact that each of these measurements are probing different aspects of the underlying biological dynamics.

The TCID_50_ assay used for dataset 1 appears to produce infectious concentrations that are shifted approximately a few orders of magnitude higher, relative to the results from the other two TCID_50_ assays. In contrast, the rRT-PCR assays seem to produce relatively consistent results across all four datasets. Also, infectious viral load appears to peak approximately 1 day later in dataset 1 compared with datasets 2–4.

### Comparison of Parameter Estimates from the Two Models

**Figure 3:**
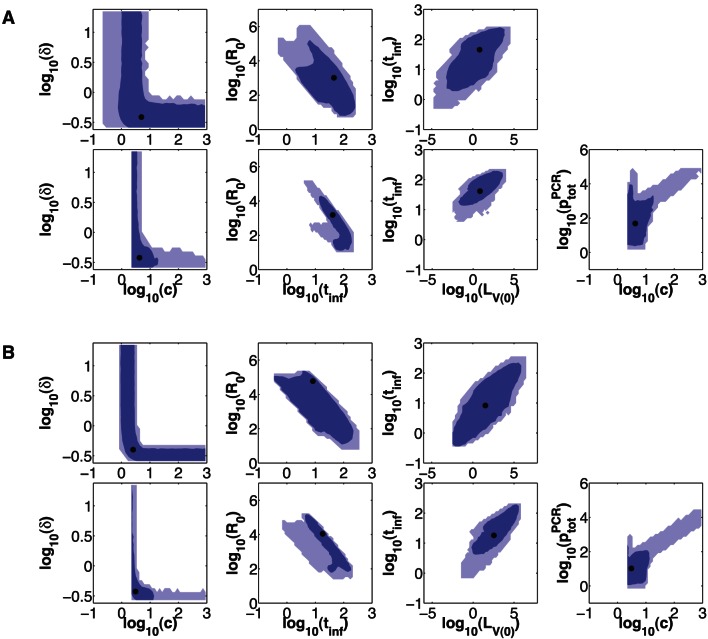
LCRs for the two Naive experiments. (**A**): LCRs obtained by fitting the single-measurement model (first row) or dual-measurement model (second row) to the combined data from dataset 1. For each model, we plot best-fit parameter estimates (dots), as well as 2-dimensional projections of the 68% LCR (inner contours) and 95% LCR (outer contours). (**B**): Same as (A), except that these LCRs were obtained by fitting each model to dataset 2.

**Figure 4:**
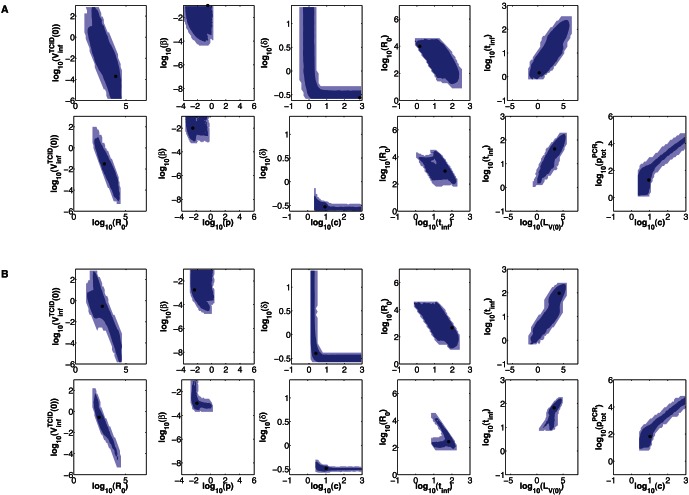
LCRs for the two PBS+IFA experiments. Same as Figure 3, except that these LCRs were obtained by fitting each model to (**A**): dataset 3, and (**B**): dataset 4. Also, we include projections onto TCID_50_ assay-dependent parameters (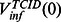, 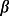, and 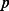) in this figure, as estimates for those parameters are able to be compared across datasets 3 and 4 (because all TCID_50_ data were obtained from the same assay).

In order to examine and compare uncertainties in parameter estimates from each model, we estimate LCRs using Equation 14. 68% and 95% LCRs for the single- and dual-measurement models fitted to each of the four different datasets are plotted in Figures 3 and 4.

In these figures, 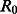 is the basic reproductive number, defined as the average number of cells that will become infected following the introduction of a single infected cell into a population composed entirely of susceptible cells [3], [9]:
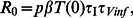
(19)where 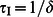 is the expected lifetime of a productively infected cell, and 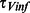 is the expected lifetime of an infectious virion (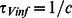 for the single-measurement model; 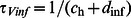 for the dual-measurement model). Also, we define the *initial number of infected cells* (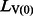) to be the average number of cells that become (latently) infected by the initial viral inoculum. Like 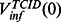, the biological interpretation of 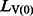 only holds in cases where ferrets were indeed infected at 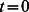. Since 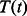 may be assumed to be approximately constant (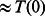) immediately following infection, we estimate 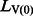 using:



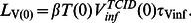
(20)If we assume that the average number of infectious virions required to infect a target cell is the same for each experiment, then 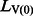 is proportional to the number of infectious virions in the initial viral inoculum, with a constant of proportionality that is identical regardless of TCID_50_ assay variability. Thus, estimates of 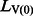 can be compared across all four datasets, unlike estimates of 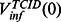. We also define an *infecting time* [10], [34]:
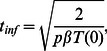
(21)as the expected time taken for a single productively infected cell to (latently) infect a second cell, when introduced into an entirely susceptible population. This physical interpretation of 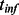 emerges from the single-measurement model if viral clearance (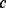) is neglected [10], and from the dual-measurement model if both host-driven clearance (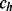) and infectious virus degradation (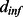) are neglected. Lastly, we define a production rate of total vRNA, 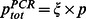, which has units 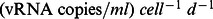.

Figures 3 and 4 show that LCRs obtained using the dual-measurement model are smaller than those obtained using the single-measurement model, for all four datasets, at both the 68% and 95% confidence levels. Best-fit parameter estimates are generally similar for the two models.

Correlations between some parameter estimates are evident in Figures 3 and 4. For example, parameter estimates for 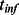 and 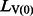 are generally correlated for both models, as large 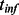 estimates generally require large 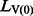 estimates in order to fit the data, while small 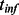 estimates are generally associated with small 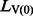 estimates. 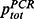 and 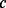 parameter estimates in the dual-measurement model are also generally correlated, and similar correlations are present in both 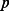 versus 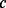 and 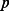 versus 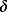 LCR projections (data not shown). In contrast, parameter estimates of 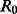 and 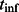, as well as 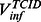 and 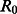, are generally anti-correlated in both models (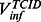 and 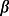 are similarly anti-correlated; data not shown).

Estimates of the 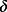 and 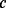 parameters display degeneracy in both models, although this is more prevalent in the single-measurement model, particularly for the fits to datasets 3 and 4. When 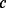 estimates are small, variations in 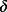 estimates do not significantly affect the goodness of fit, and vice versa. The 68% and 95% confidence regions for 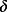 and 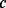 are unbounded for all four single-measurement model fits, unlike in the dual-measurement model where the equivalent parameters, 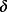 and 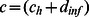, are bounded in some cases; e.g. datasets 3 and 4.

**Figure 5:**
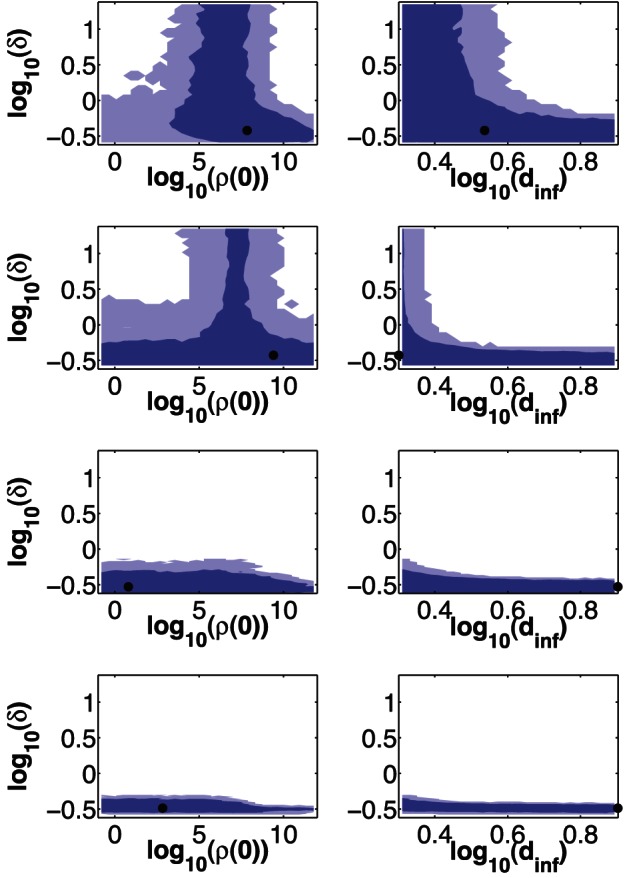
LCRs for the dual-measurement model fitted to datasets 1–4. LCRs obtained by fitting the dual-measurement model to combined data; from dataset 1 (first row) to dataset 4 (fourth row).

The parameters 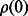 and 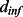 (not represented in Figures 3 and 4) have unbounded confidence regions for all dual-measurement model fits. Figure 5 shows LCR projections of 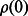 or 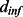 versus 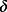 for all four datasets. For LCR projections of 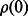 or 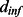 versus model parameters other than 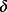, there is generally little or no correlation evident (data not shown).

**Figure 6:**
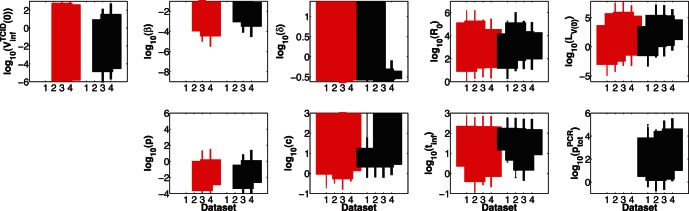
Parameter estimates. Best-fit estimates (dots), 68% CIs (thick error bars) and 95% CIs (thin error bars) are shown for each parameter, for fits of the single-measurement model (red) or dual-measurement model (black) to each dataset.

Table 3 and Figure 6 show best fit parameter estimates and CIs, for each model fitted to each dataset. We can only compare parameter estimates across different datasets if those parameters do not have 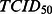 in their units (i.e. 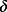, 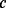, 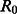, 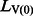, 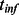, and 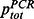), due to the aforementioned TCID_50_ assay variability (see “Fitting the data”). This restriction does not apply to comparisons between datasets 3 and 4, as the same TCID_50_ assay was used for both datasets. Indeed estimates for all parameters are consistent between these two datasets, for both the single- and dual-measurement models.

**Table 3:** Parameter estimates.

	Single-measurement model			Dual-measurement model					
Dataset	1	2	3		4	1	2	3	4
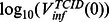	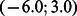 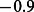	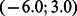 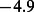	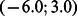 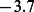		 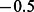	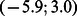 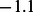	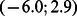 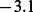	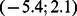 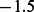	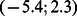 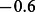
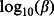	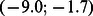 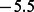	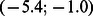 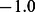	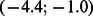 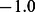		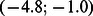 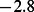	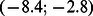 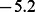	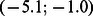 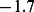	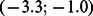 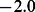	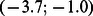 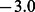
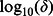	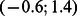 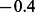	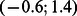 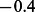	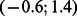 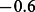		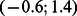 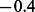	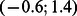 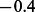	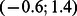 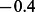	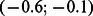 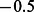	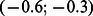 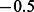
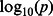	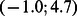 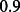	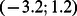 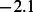	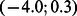 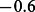		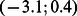 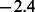	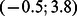 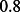	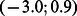 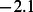	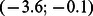 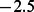	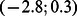 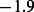
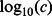	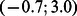 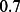	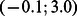 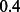	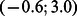 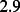		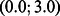 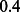	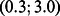 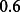	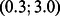 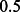	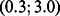 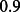	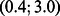 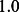
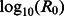	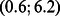 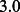	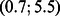 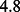	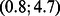 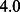		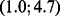 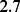	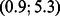 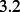	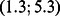 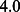	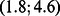 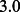	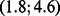 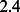
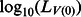	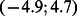 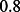	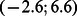 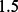	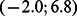 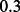		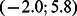 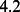	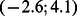 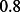	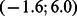 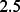	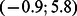 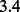	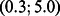 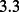
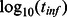	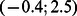 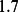	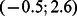 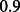	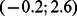 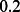		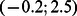 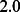	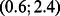 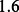	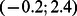 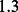	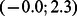 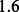	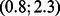 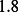
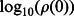	−	−	−		−	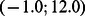 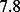	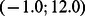 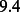	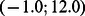 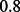	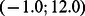 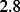
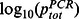	−	−	−		−	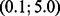 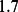	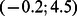 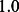	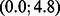 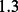	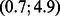 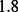
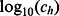	−	−	−		−	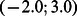 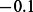	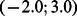 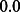	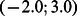 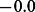	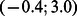 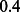
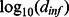	−	−	−		−	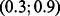 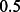	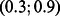 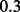	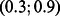 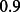	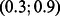 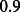
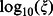	−	−	−		−	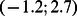 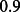	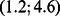 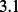	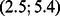 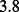	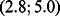 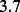

For each parameter, we show the best-fit estimates (1st row) as well as 95% CIs (2nd row; in parentheses) for each model fit to each dataset.

Best-fit estimates for 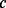, 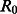, 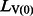, and 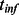 are more consistent across the different datasets for the dual-measurement model, compared with the single-measurement model. The range of best-fit estimates for these parameters are, for the dual- and single-measurement models, respectively: 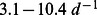 and 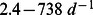 for 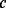; 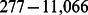 and 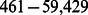 for 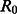; 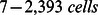 and 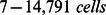 for 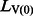; and 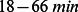 and 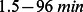 for 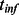. However, the 95% CIs for 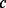, 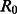, 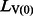, and 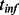 all cover a relatively large range for both models, although again this range is smaller for the dual-measurement model than for the single-measurement model. For best-fit estimates of 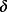, the variability across different datasets is similar for both models. For each model, 68% and 95% CIs for 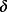, 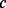, 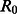, 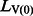, and 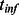 are self-consistent across all four datasets, and this is also true for 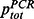 in the dual-measurement model.

All CIs from the dual-measurement model are either similar in size or smaller than those from the single-measurement model. The most prominent reduction in uncertainty between the two models is seen in estimates of 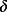 (at the 95% confidence level for datasets 3 and 4) and 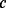 (in 68% CIs for datasets 1 and 2). For any given dataset, all CIs are consistent between the two different models.

## Discussion

### Main Findings

Our analyses demonstrate that the ratio of total (rRT-PCR) to infectious (TCID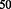) viral particle concentration is time-dependent in acute influenza infection of ferrets. Thus the biological processes underlying *in vivo* infection can potentially be probed more comprehensively by including both measurements in a within-host model. Fitting such a model allows most parameters to be estimated with reduced uncertainty (smaller LCRs), but this is not the case for all parameters. Also, best-fit estimates for 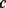, 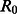, 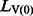, and 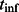 are more consistent across datasets for such a model, although large CIs in parameter estimates mean that this result is not well supported statistically.

The observed time-dependence in the ratio of total to infectious virus, 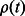 (Figure 2), can potentially be explained within the context of the dual-measurement model, in terms of three distinct phases:

1. During the first few (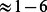) hours of infection, 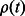 increases briefly as there are no productively infected cells in the dual-measurement model initially, and we have assumed that infectious virus decays faster (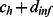) than total virus (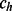).
2. During the phase of exponential viral growth, 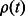 tends towards a value that is just above 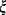, as viral production from infected cells becomes the main contributor to 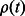. Since infectious virus decays faster than total virus in the dual-measurement model, 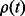 must tend towards a value that is greater than 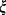.
3. After the exponential growth phase (around the viral load peak), the model transitions into a phase of exponential viral decay, dominated by viral clearance and/or degradation (loss of infectivity). As infectious virus decays faster than total virus in the model, 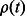 increases during this phase.

Another possible explanation for the time-dependence of 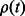 has been investigated by Vaidya *et al.* [32] within the context of *in vivo* simian immunodeficiency virus (SIV) infection, by allowing the infectivity rate in a within-host model to vary with time. Vaidya *et al.* also discussed alternative mechanisms for generating time-dependence in 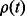 during SIV infection, which could potentially apply to *in vivo* influenza infection as well – these include a time-varying production rate for infectious virions, and the coating of infectious virions by antibody.

We observed that the infectious viral load in dataset 1 appears to have a delayed peak relative to that in datasets 2–4 (Figure 2). Such a delay might arise as a consequence of using two different tests (rRT-PCR or a rapid test) to determine the time that each ferret was co-housed with the next ferret in the serial passage line (see “Ferret experimental data”). When the rapid test was used (datasets 3 and 4), ferrets were more likely to have a higher viral load upon being co-housed with the next ferret in line, relative to when rRT-PCR was used (datasets 1 and 2), due to the higher sensitivity of the rRT-PCR assay (data not shown). Consequently, ferrets in datasets 1 and 2 may have been more likely to become infected either relatively late, or with a relatively low initial viral inoculum, or both, compared with ferrets in datasets 3 and 4. The greater the number of ferrets with late infection time and/or low initial viral load, the more likely it is that their combined data will have a delayed viral load peak. Due to stochastic variation, it is possible that this occurred more frequently for ferrets in dataset 1 compared with dataset 2.

Subsequently, it is interesting that best-fit estimates from the dual-measurement model of the initial number of infected cells, 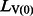, for datasets 1 and 2 are approximately 1–2 orders of magnitude lower (≈ 10−severalhundred *cells*) than those for datasets 3 and 4 (≈ severalthousand *cells*). This could point towards a relatively low initial viral inoculum and/or relatively late time of infection for the combined data in each of datasets 1 and 2, consistent with the potential causes of a delayed viral load peak discussed above. However, we must keep in mind that the biological interpretation of 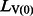 only applies in cases where ferrets were indeed infected at 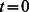. Also, we cannot make a statistically significant inference regarding differences in 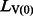 estimates between the different datasets, as the 68% and 95% uncertainties of these estimates all overlap. Indeed, parameter estimate uncertainties, for all parameters that do not contain 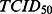 in their units, are self-consistent across all four datasets. Nonetheless, the potential to compare 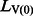 across different datasets (with infectivity data originating from different TCID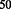 assays) highlights the usefulness of estimating 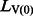 in addition to the 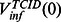 parameter, which cannot be compared across datasets that use different infectivity assays.

We found that estimates of certain parameters are correlated, for both models, while certain other parameter estimates are anti-correlated (Figures 3 and 4). Such correlations can arise when fitting data due to mechanistic interrelationships between model parameters. For instance, 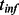 and 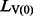 estimates were generally correlated with each other because decreasing 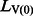 delays the increase in viral load – this change in viral load dynamics can be compensated for by increasing the rate of spread of infection (e.g. by decreasing 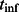). An analogous interrelationship applies to *increases* in 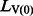 and 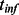. Importantly, investigating such correlations between parameters using LCR projections can provide insight into how parameter estimation could potentially be improved. For instance, the anti-correlation between 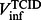 and 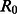 indicates that any attempt to improve estimates of 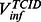 (for example, by measuring viral load more frequently close to the time of infection) could have the added benefit of generating stronger estimates of 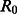.

We also observed degeneracy between estimates of the 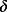 and 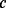 parameters, with small 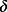 estimates associated with degeneracy in 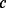, and vice versa (Figures 3 and 4). This degeneracy is not unexpected based on previous analytic results for a single stage model that showed that the post-peak decay rate of infectious viral load is governed by the smallest of the 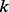, 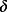, and 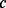 parameters [18].

We found that, despite confidence regions being unbounded for 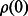 and 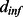, potentially useful information can still be obtained by investigating LCR projections for these parameters (Figure 5). For fits to datasets 1 and 2, the LCR projections in Figure 5 indicate that if 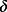 were to be measured independently, and if its estimated value were ≳2, the identifiability of 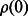 and 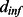 could potentially be improved. However, this is not the case for fits to datasets 3 and 4, as little correlation is evident in those LCR projections. There is generally little or no correlation evident when 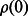 or 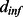 are plotted against most other model parameters.

We compare parameter estimates obtained when modelling ferret infection data with the dual-measurement model (Table 3), to those obtained when modelling *in vitro* influenza data [10], [36], and *in vivo* data from humans [3]–[5] and mice [17], [19]. Unfortunately, we cannot compare estimates of 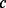, 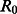, 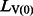, and 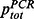 to those from other studies, because our estimates all have large (and sometimes unbounded) 95% CIs. We only compare estimates of 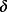 and 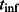, as those are the only remaining parameters that do not contain 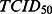 in their units. Acknowledging potential variation in biological parameters by strain, our best-fit estimates of 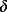 (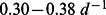; 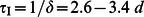) are slightly larger than 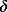 estimates from several *in vitro* [36] and *in vivo* [3]–[5], [19] modelling studies (which are roughly in the range 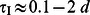) as well as estimates from direct experimental measurements of the average lifetime of influenza-infected cells (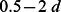; as reviewed by Beauchemin & Handel [7]), but are in agreement with the *in vivo* estimates of Miao *et al.* [17] (which are in the range 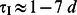). For 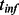, our best-fit estimates (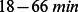) and 95% CIs (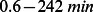) are consistent with previous *in vitro* estimates [10], [36] (which are roughly in the range 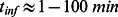).

### Main Limitations of this Study

Both the single- and dual-measurement models we use are target cell-limited – i.e. the progress of the infection is limited only by the availability of susceptible epithelial cells, rather than by any form of time-varying immune response. Although such models can generate viral load dynamics that are consistent with *in vivo* data, it is likely that immune response dynamics contribute towards limiting the spread of infection (recent reviews [6]–[8] discuss evidence for the importance of immune responses in regulating influenza dynamics). Several recent within-host modelling papers have required the inclusion of some form of time-varying immune response in their models in order to adequately explain the dynamics of both viral load and immune response data [14], [16], [17]. The *in vivo* experiments analysed in this paper, however, did not include regularly sampled measurements of immune responses and thus these recent techniques are not able to be applied here.

While confidence regions for parameters have been substantially reduced with the dual-measurement model (Figures 3 and 4), parameter estimates do remain somewhat poorly constrained, reflecting fundamental limitations in the inferences we can draw from routinely available viral load data. Confidence regions for 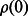 and 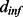 are unbounded for fits of the dual-measurement model to all four datasets. For 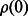, this is likely a consequence of a lack of data within the first 24 hours of infection, coupled with the fact that the dual-measurement model can generate 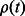 dynamics that are similar to those seen in the data, for many different values of 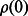. For 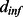, confidence regions are unbounded because the range we restrict 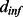 estimates to is very small (Text S1). It may be possible to alleviate this identifiability issue for 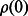 in future experiments, by taking more frequent measurements around the time of infection. Also, unboundedness in 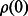 estimates should be less of a problem in any experiment where animals are inoculated rather than naturally infected, as 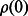 could be measured in the inoculum.

Because all ferrets were naturally infected, the exact time of infection for each ferret is unknown. This affects the physical interpretation of the 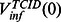, 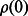, and 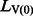 parameters, in any cases where ferrets did not become infected at approximately the time we have assumed. This issue could potentially be mitigated in future experiments, by reducing the duration that each infected animal is exposed to susceptible animals to a single, brief period of exposure. This parameter interpretation problem does not apply to experiments where animals are inoculated rather than naturally infected.

When constructing the dual-measurement model, we assumed that host-driven clearance of both infectious and non-infectious viral particles occurred at the same rate, consistent with several models of HIV infection [37], [38]. However, there is currently a lack of experimental evidence that tests the biological validity of this assumption. If models similar to the dual-measurement model are to be used in future, it will be important to investigate the relative clearance rates of infectious and non-infectious viral particles further.

In this work, total viral particles were defined as particles that contain vRNA measurable via rRT-PCR. However, rRT-PCR measurements may underestimate the concentration of non-infectious (and hence total) virus, because non-infectious particles may contain incomplete vRNA [21]. Any non-infectious particles that were missing the portion of influenza A matrix 1 gene used here to amplify vRNA during rRT-PCR assays, would not have been detectable (Guarnaccia *et al.*, under review).

Further, it is likely that the different subpopulations of virus particles in an *in vivo* influenza infection are more diverse and complex than the two subpopulations (infectious and total virus) included in the dual-measurement model. Richer classifications of influenza subpopulations have previously been investigated *in vitro*. For example, Marcus *et al.* [20] studied the dynamics of plaque-forming particles, defective interfering particles, non-infectious cell-killing particles, and hemagglutinating particles during *in vitro* serial passaging. Within-host modelling of the dynamics of such viral particle subpopulations has the potential to increase our understanding of influenza pathogenicity and immune responses. Our dual-measurement model provides an incremental step towards capturing the complex *in vivo* dynamics of these virus particle subpopulations.

### Ramifications of Our Findings

Recently, some influenza modelling studies have implicitly assumed that the *in vivo* ratio of total to infectious virus is constant over time [15], [22]–[24]. However, results from other *in vivo* influenza studies suggest that this ratio is time-dependent (e.g. [25]–[28]), and this is also supported by the data analysed in this work.

Our results also highlight how variation in TCID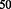 assay sensitivity and calibration may hinder model interpretation, as we were unable to compare estimates for any parameters with 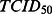 in their units. Future improvements in infectivity assay reproducibility will greatly aid the capability to compare parameter estimates across different studies. For example, development of an international standard stock of influenza virus could provide inter-laboratory calibration of infectivity assays, analogous to the international standardisation of hemagglutination-inhibition and virus neutralisation assays [42].

Recent reviews of within-host influenza modelling have discussed the need for more comprehensive datasets, in order to obtain a more accurate picture of infection dynamics and enhance the precision of biological inferences based on within-host modelling [7], [8]. The techniques outlined in this work can be used to increase the diversity of available data in order to further inform such model-based biological inferences.

## Supporting Information

Text S1 **A comparison of previous estimates of viral clearance rate and viral degradation rate, as well as further details regarding data fitting.**
(PDF)
