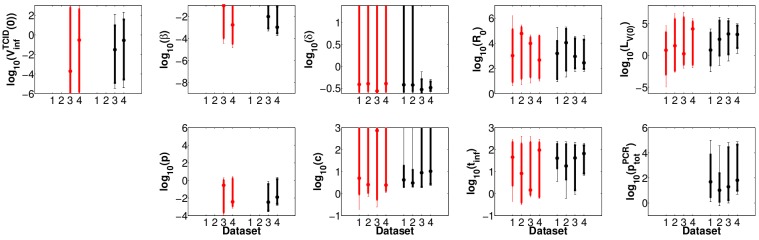# Correction: Reducing Uncertainty in Within-Host Parameter Estimates of Influenza Infection by Measuring Both Infectious and Total Viral Load

**DOI:** 10.1371/annotation/3b815950-b0eb-4aac-9a83-e92f830f844b

**Published:** 2013-11-07

**Authors:** Stephen M. Petrie, Teagan Guarnaccia, Karen L. Laurie, Aeron C. Hurt, Jodie McVernon, James M. McCaw

In Table 1, an error occurred in the variable "V" in the fourth row. Please see the correct Table 1 here: 

**Figure:**
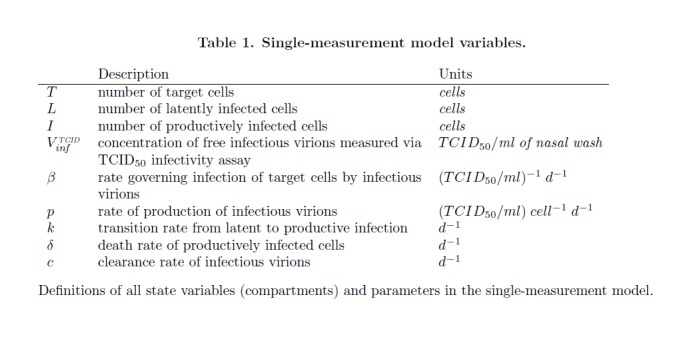


In Table 3, the best-fit estimates have been printed to the left of the 95% CIs. The missing CIs for Row 1 in the "Single-measurement model" Dataset 4 are (-6.0;3.0). Please see the correct Table 3 here: 

**Figure:**
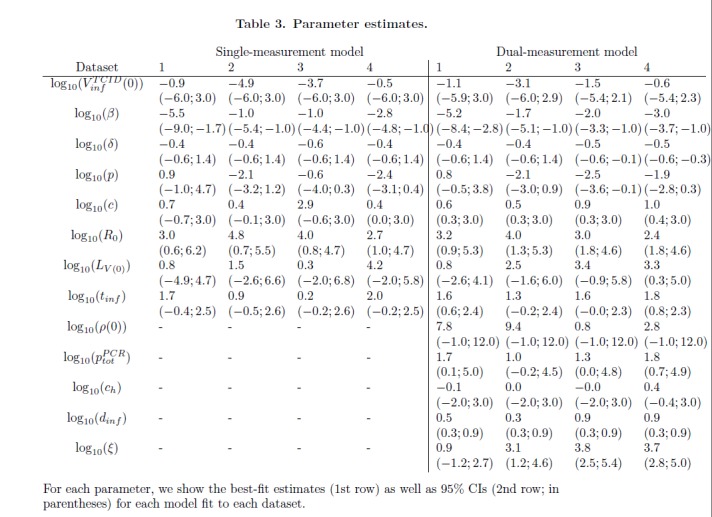


In Figure 2, the line weights were incorrectly altered. Please see the correct Figure 2 here: 

**Figure:**
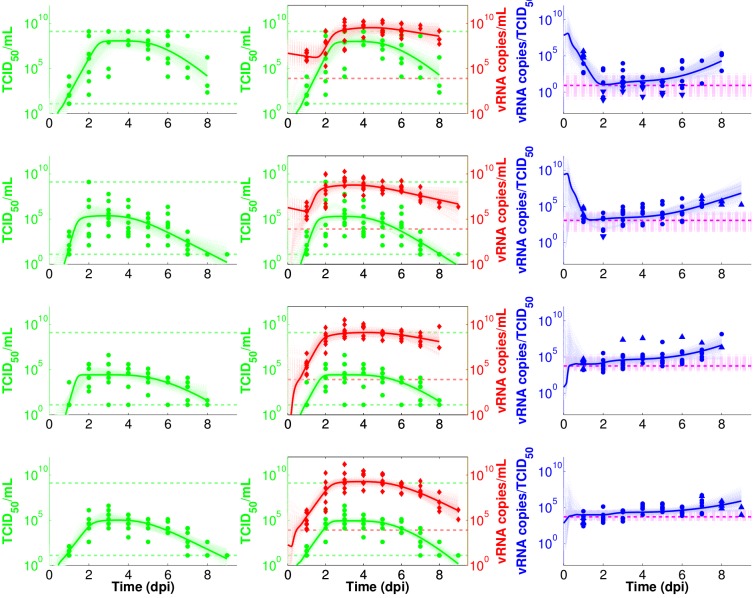


In Figure 6, the error bars in the graphs were incorrectly merged. Please see the correct Figure 6 here: 

**Figure:**