# Supplementary material for: Reducing Uncertainty in Within-Host Parameter Estimates of Influenza Infection by Measuring Both Infectious and Total Viral Load
Source: PLoS One. 2013 May 15;8(5):e64098. doi: 10.1371/journal.pone.0064098 (PMC3655064; doi:10.1371/journal.pone.0064098)
Supplement: Text S1 — A comparison of previous estimates of viral clearance rate and viral degradation rate, as well as further details regarding data fitting. (PDF) [file pone.0064098.s001.pdf]

Supporting information

Comparing previous estimates of viral clearance rate and viral degradation rate

Here we investigate the biological plausibility of the $c_h \gg d_{ni}$ assumption, by comparing previous estimates of the viral clearance rate to estimates of the viral degradation rate, for various strains of influenza A:

- Estimates of c from within-host modelling of *in vivo* data range from 1.4 d^{-1} ($1/c = 17$ h) to 28.4 d^{-1} ($1/c = 50.7$ min) [1–5]. This range does not include estimates of c from *all* models within these references, as several of those models explicitly include some form of time-varying immune response in addition to the c parameter. As these studies did not estimate either c_h or d_{inf} directly, we treat each estimate of c as an upper bound on the corresponding estimate of c_h.

- Daum *et al.* [6] measured virus degradation via rRT-PCR assays — their results suggest a degradation rate of approximately 0.06–0.19 d^{-1}. We do not include their results for influenza stored in viral transport medium (VTM), as Daum *et al.* stated that those results suggested detection of vRNA via rRT-PCR was hindered for virus stored in VTM. Wang *et al.* [7] found that there was no significant change in rRT-PCR viral concentration when virus was stored for up to 3 days at room temperature in various storage media. Schulze-Horsel *et al.* [8] measured d_{ni} *in vitro* using HA assays (which measure viral concentration via the hemagglutinin surface protein rather than via internal vRNA), and found values of 0 d^{-1}, 0 d^{-1}, and 0.24 d^{-1} for three different strains of influenza. Möhler *et al.* [9] measured the degradation rate of influenza (via HA assays) to be 0.072 d^{-1}.

These estimates support the biological plausibility of the $c_h \gg d_{ni}$ assumption.

Further details regarding data fitting

Fixed parameter and biologically realistic ranges for fitted parameters

We fix $k = 3$ d^{-1}, consistent with estimates obtained when fitting models (with normal or log-normal delay distributions for L and I) to *in vitro* data [4, 10]. We investigated fitting for the latent period, k, but found that not only was k unidentifiable in both models, fitting rather than fixing k also significantly reduced model inference capability for several other parameters (data not shown).

For all fitted parameters, we specify a range of biologically plausible values (Table S1) which restrict the parameter space searchable by the genetic algorithm.

Estimating a lower bound for $V_{inf}^{TCID}(0)$

Theoretically, influenza infection could be initiated by as little as a single infectious virion in the URT. In order to set the lower bound for $V_{inf}^{TCID}(0)$ to a value that corresponds to 1 infectious virion in the URT, we need to estimate:

1. the number of infectious virions that correspond to 1 $TCID_{50}$ (Handel *et al.* [2] estimated this to be somewhere in the range 1-100),

2. the conversion factor between *infectious virions/ml* in the URT and *infectious virions/ml* in nasal wash samples when they are assayed (Handel *et al.* [2] estimated that nasal wash sample concentrations were somewhere in the range of 1-100 times smaller than corresponding URT concentrations, while Beauchemin *et al.* [11] found that if a sample was frozen and then thawed before being assayed, there was about a 10-fold reduction in infectious viral load as measured via plaque assay),

3. the volume of the ferret URT (we estimate this to be approximately 1 *ml* based on URT volumes [12] of mammals with similar body weight to the ferrets in the Guarnaccia *et al.* experiments; i.e. 500–1500 *g*).

Based on these rough estimates, we assume a conservative lower bound of 10^{-6} $TCID_{50}/ml$ for $V_{inf}^{TCID}(0)$.

References

1. Baccam P, Beauchemin CAA, Macken CA, Hayden FG, Perelson AS (2006) Kinetics of influenza A virus infection in humans. Journal of Virology 80: 7590–9.

2. Handel A, Longini IM, Antia R (2007) Neuraminidase inhibitor resistance in influenza: assessing the danger of its generation and spread. PLoS Computational Biology 3: e240.

3. Miao H, Hollenbaugh JA, Zand MS, Holden-Wiltse J, Mosmann TR, et al. (2010) Quantifying the early immune response and adaptive immune response kinetics in mice infected with influenza A virus. Journal of Virology 84: 6687–98.

4. Holder BP, Beauchemin CAA (2011) Exploring the effect of biological delays in kinetic models of influenza within a host or cell culture. BMC Public Health 11 Suppl 1: S10.

5. Smith AM, Adler FR, McAuley JL, Gutenkunst RN, Ribeiro RM, et al. (2011) Effect of 1918 PB1-F2 Expression on Influenza A Virus Infection Kinetics. PLoS Computational Biology 7: e1001081.

6. Daum LT, Worthy SA, Yim KC, Nogueras M, Schuman RF, et al. (2010) A clinical specimen collection and transport medium for molecular diagnostic and genomic applications. Epidemiology and Infection : 1–10.

7. Wang X, Zoueva O, Zhao J, Ye Z, Hewlett I (2011) Stability and infectivity of novel pandemic influenza A (H1N1) virus in blood-derived matrices under different storage conditions. BMC Infectious Diseases 11: 354.

8. Schulze-Horsel J, Schulze M, Agalaridis G, Genzel Y, Reichl U (2009) Infection dynamics and virus-induced apoptosis in cell culture-based influenza vaccine production-Flow cytometry and mathematical modeling. Vaccine 27: 2712–22.

9. Möhler L, Flockerzi D, Sann H, Reichl U (2005) Mathematical model of influenza A virus production in large-scale microcarrier culture. Biotechnology and Bioengineering 90: 46–58.

10. Holder BP, Simon P, Liao LE, Abed Y, Bouhy X, et al. (2011) Assessing the in vitro fitness of an oseltamivir-resistant seasonal A/H1N1 influenza strain using a mathematical model. PloS ONE 6: e14767.

11. Beauchemin CAA, McSharry JJ, Drusano GL, Nguyen JT, Went GT, et al. (2008) Modeling amantadine treatment of influenza A virus in vitro. Journal of Theoretical Biology 254: 439–51.

12. Ménache MG, Hanna LM, Gross EA, Lou SR, Zinreich SJ, et al. (1997) Upper Respiratory Tract Surface Areas and Volumes of Laboratory Animals and Humans: Considerations for Dosimetry Models. Journal of Toxicology and Environmental Health, Part A 50: 475–506.

13. Smith AM, Perelson AS (2011) Influenza A virus infection kinetics: quantitative data and models. Wiley Interdisciplinary Reviews: Systems Biology and Medicine 3: 429–445.

14. Beauchemin CAA, Handel A (2011) A review of mathematical models of influenza A infections within a host or cell culture: lessons learned and challenges ahead. BMC Public Health 11 Suppl 1: S7.

15. Saenz RA, Quinlivan M, Elton D, Macrae S, Blunden AS, et al. (2010) Dynamics of influenza virus infection and pathology. Journal of Virology 84: 3974–83.

16. Pawelek KA, Huynh GT, Quinlivan M, Cullinane A, Rong L, et al. (2012) Modeling within-host dynamics of influenza virus infection including immune responses. PLoS Computational Biology 8: e1002588.

17. Arndt U, Wennemuth G, Barth P, Nain M, Al-Abed Y, et al. (2002) Release of macrophage migration inhibitory factor and CXCL8/interleukin-8 from lung epithelial cells rendered necrotic by influenza A virus infection. Journal of Virology 76: 9298.

18. Zhirnov O, Klenk HD (2003) Human influenza A viruses are proteolytically activated and do not induce apoptosis in CACO-2 cells. Virology 313: 198–212.

19. Horsfall FL (1954) On the reproduction of influenza virus. Quantitative studies with procedures which enumerate infective and hemagglutinating virus particles. The Journal of Experimental Medicine 100: 135–161.

20. Paucker K, Henle W (1955) Studies on host-virus interactions in the chick embryo-influenza virus system. XI. The effect of partial inactivation of standard seed virus at 37C upon the progeny. The Journal of Experimental Medicine 101: 479–492.

21. Gausch CR, Smith TF (1968) Replication and plaque assay of influenza virus in an established line of canine kidney cells. Applied Microbiology 16: 588–94.

22. Orthel FW (1972) Influenza virus titrations and the inhibitor of hemagglutination in normal allantoic fluid. Archiv für die gesamte Virusforschung 38: 347–56.

Tables

Table S1. Biologically realistic parameter ranges.

Parameter	Bounds	Source[a]
$V_{inf}^{TCID}(0)$	$[10^{-6}, 10^{3}]$	Upper bound informed by initial V_{inf}^{TCID} measurements within the Guarnaccia *et al.* (under review) data; lower bound based on an estimate of the lowest possible TCID_{50} value that corresponds to a single infectious virion in the URT (see "Estimating a lower bound for $V_{inf}^{TCID}(0)$").
$\rho(0)$	$[10^{-1}, 10^{12}]$	Based on the variability of $\rho(t)$ within the Guarnaccia *et al.* (under review) data[b].
β	$[10^{-9}, 10^{-1}]$	Previous *in vivo* and *in vitro* modelling estimates of β [1–3, 5, 8, 9, 11, 15, 16].
δ	$[0.24, 24]$	This range corresponds to average productively infected cell lifetimes (i.e. $1/\delta$) from 1 h to 100 h, consistent with previous *in vitro* observations [9, 17, 18] as well as both *in vivo* and *in vitro* model-fitting estimates [1–5, 8, 9, 11].
p	$[10^{-6}, 10^{6}]$	Previous *in vivo* and *in vitro* modelling estimates of p [1–5, 8, 9, 11, 15, 16].
c	$[10^{-1}, 10^{3}]$	Previous *in vivo* estimates for c obtained from target cell-limited models in which infection progress is limited by the availability of susceptible cells, rather than by immune response dynamics [1–5].
c_h	$[10^{-2}, 10^{3}]$	Previous *in vivo* estimates for c obtained from target cell-limited models in which infection progress is limited by the availability of susceptible cells, rather than by immune response dynamics [1–5][c].
d_{inf}	$[2, 8]$	Previous *in vitro* estimates of d_{inf} [8, 10, 11, 19–22].
ξ	$[10^{-2}, 10^{7}]$	Based on the variability of $\rho(t)$ within the Guarnaccia *et al.* (under review) data.

Lower and upper bounds for each fitted parameter.

[a] For reviews of parameter estimates obtained by within-host modelling influenza studies, see [13, 14].

[b] We use an upper bound that is several orders of magnitude higher than the highest measured $\rho(t)$ value in the data, in order to allow for the possibility that many infectious virions could become non-infectious during transmission from one ferret to another.

[c] We use a smaller lower bound for c_h than that used for c, as we expect c_h to be somewhat lower than previous estimates of c, since those estimates implicitly include both c_h and d_{inf}.
